# Characterization of a newly isolated phage infecting pathogenic *Escherichia coli* and analysis of its mosaic structural genes

**DOI:** 10.1038/s41598-018-26004-4

**Published:** 2018-05-24

**Authors:** Qin Peng, Yihui Yuan

**Affiliations:** 1 Ministry of Education Key Laboratory for Ecology of Tropical Islands, College of Life Sciences, Hainan Normal University, Haikou, 571158 P. R. China; 2 State Key Laboratory of Marine Resource Utilization in South China Sea, Hainan University, Haikou, 570228 China

## Abstract

The outbreak of multidrug-resistant pathogenic bacteria made the discovery of novel control strategies necessary. Phages have regained attention for their specific lytic activity against pathogenic bacterium. A newly isolated phage infecting the clinical *Escherichia coli* isolates, including several multidrug-resistant strains, was isolated, and this phage showed high control effects against the tested pathogenic *E*. *coli* strains. Host range analysis revealed that although the phage exhibited broad lytic spectrum against the tested *E*. *coli* strains, it could not lyse strains from the other species. Comparative genomic analysis showed that phages had undergone at least three genome recombination events during the evolutionary process at the position of the three phage tail genes, which was reported to be associated with the host range determination of the phage. The recombinant tail proteins contained functional domains that were highly similar with genes of the *Salmonella* phage and genes of *Pseudomonas* and *Neisseria*. The findings of this study not only provide resources for developing phage therapy against *E*. *coli*, but also showed the highly variable genome structure of the phage.

## Introduction

*Escherichia coli* is a type of Gram-negative bacterium of the *Enterobacteriaceae* family, the majority of which reside in the human gastrointestinal tract as a part of the normal flora. However, some *E*. *coli* have evolved into virulent straains, and are responsible for a variety of diseases, including intestinal diarrhea, urinary tract infections (UTI), septicemia, pneumoniae, and meningitis^1,2^. Like the other widely distributed multidrug-resistant pathogenic bacterial species, the multidrug resistance of *E*. *coli* has high prevalence.

The development of multidrug-resistant *E*. *coli* gained extra concern with respect to human health, and infections caused by *E*. *coli* are usually harder to treat, resulting in increased severity and duration of infection. Production of extended-spectrum β-lactamases (ESBL), found in some strains of *Enterobacteriaceae*, are one of the resistant mechanisms, which act as a worldwide threat. These species are chief culprits that cause treatment failure, including infections of the urogenital tract, abdomen, and bloodstream^3^. In addition to the production of ESBL, these strains are prevalently related to other mechanisms of resistance, making them multidrug resistant (MDR)^4^. The development of antimicrobial resistance can occur via two processes: mutation in certain genes and acquisition of resistant genes by horizontal transfer^5^. Therefore, it can contribute to the spread of antimicrobial resistance between bacterial species as well as to other members of the same species. Globally, antibiotic resistance analysis reveals that this serious threat is no longer a worry for the future, which occurring now in every region of the world, urging the exploration of new agents to resolve this situation.

Phages have gained growing attention as an alternative candidate to antibiotics. Phages possess unique advantages, and nature provides an almost inexhaustible supply, and no two identical phages have ever been found. However, in consideration of the potential for immunogenicity, rapid toxin release by lytic action, development of bacterial resistance and other else during the use of phages for therapeutic purposes, phage therapy was still actively pursued^6,7^. For example, through shedding the receptor on the cell surface that the virus used to enter, a bacterium becomes resistant to one phage. It can be overcome by adding more phages to the viral cocktails that patients receive^8^. In previous study, the phage cocktails consisting of 10 and 16 T4 type phage isolates could lyse one half to two thirds of the *E*. *coli* strains, representing five main pathotypes isolated from diarrheal patients^9^. Galtier M. *et al*. utilized a single dose of a cocktail, which consisted of three phages, led to a sharp decrease of *E*. *coli* levels throughout the gut^10^. These studies suggest that the *E*. *col*i phage has high potential to be used in controlling pathogenic *E*. *coli*, especially multidrug resistant *E*. *coli*.

To exploit more efficient phages that target clinically relevant *E*. *coli*, a newly isolated phage named vB_EcoS_HSE2 was isolated from hospital sewage, its biological and genomic characters were investigated and its potential usage in controlling pathogenic *E*. *coli* was evaluated. The finding of this study increases our understanding on *E*. *coli* phage diversity and provides resource for controlling pathogenic *E*. *coli* infections.

## Results

### Isolation and virion morphology observation of phage vB_EcoS_HSE2

**Figure 1:**
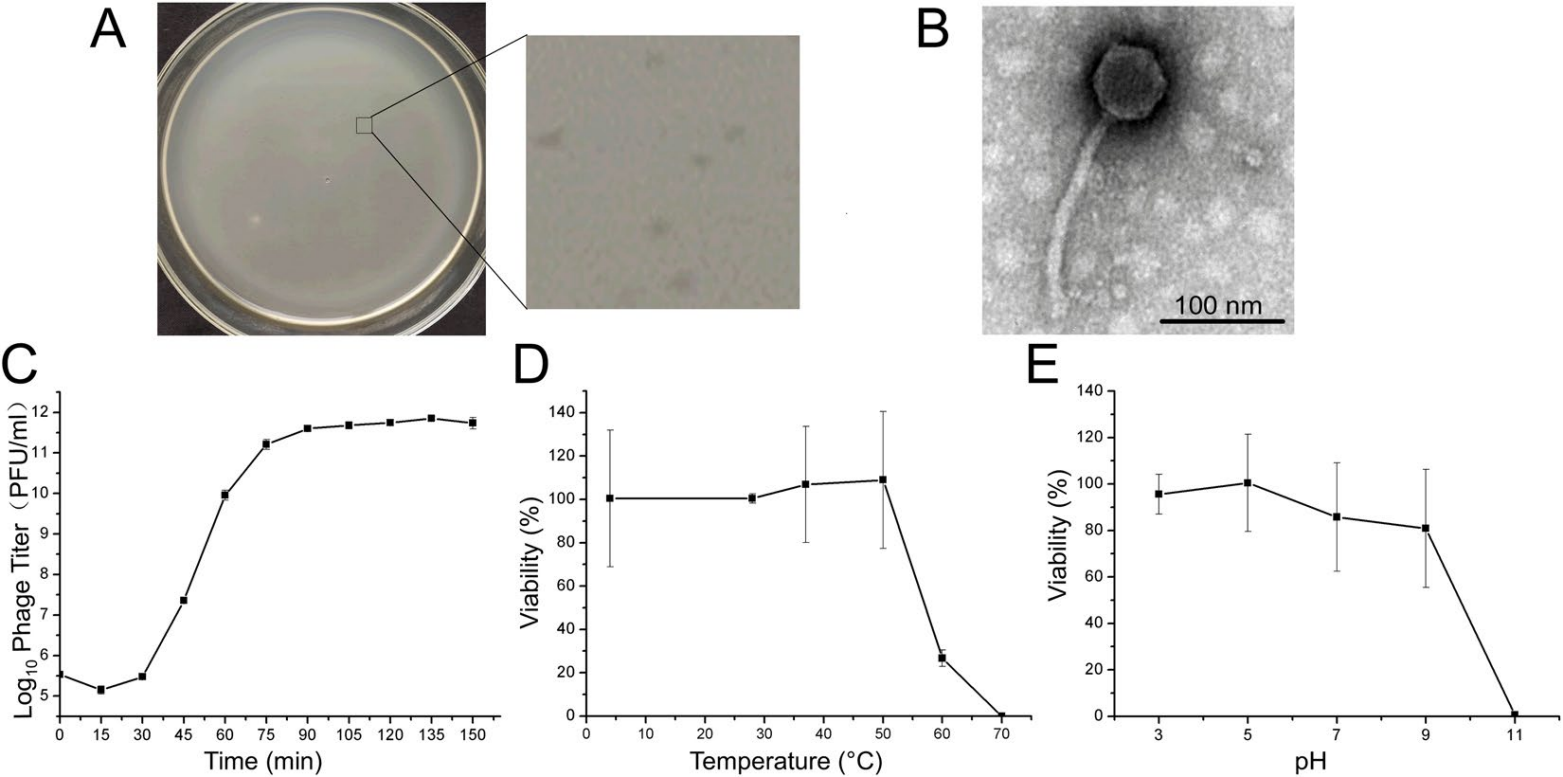
Morphology observation and characterization of phage vB_EcoS_HSE2. (**A**) Plaque morphologies of phage vB_EcoS_HSE2. (**B**) Virion morphology of phage vB_EcoS_HSE2. The phage virion was stained with potassium phosphotungstate and observed using transmission electron microscopy. (**C**) One step growth curve of the phage. (**D**) Thermal tolerance of the phage. (**E**) Tolerance of the phage to different pH treatment.

By using hospital sewage samples, one phage infecting *E*. *coli* strain 40371 was isolated, and the phage was named vB_EcoS_HSE2. Phage vB_EcoS_HSE2 formed transparent, small, round plaques of approximately 0.5 mm in diameter on the lawn of *E*. *coli* strain 40371 (Fig. 1A). TEM observation of the phage virion revealed that the phage had an icosahedral capsid with a diameter of 56 nm and a non-contractile tail that was approximately 178 nm in length and 12 nm in diameter (Fig. 1B). Based on the morphology, the phage was classified as a *Siphoviridae* family phage.

### One-step growth curve

The one-step growth curve of phage vB_EcoS_HSE2 was analyzed by infecting the exponential growth strains with the phage at an MOI of 1.0. The latent period of the phage, which was the period between the absorption of the phage to the host bacterium and the beginning of the lysis of the host bacterium, was approximately 30 min, and the final concentration of the phage reached up to 10^11^ PFU/ml (Fig. 1C). Based on the final concentration of the phage and the concentration of the bacterial cell that was infected by the phage, the calculated burst size of phage vB_EcoS_HSE2 was about 86 PFU per bacterial cell.

### Highly specific lytic spectrum

In this study, the host range of the phage vB_EcoS_HSE2 was tested against 18 clinical pathogenic strains, including 11 *E*. *coli* strains, and strains of *Pseudomonas aeruginosa*, *Salmonella enterica*, *Neisseria gonorrhoeae*, *Klebsiella pneumoniae*, *Bacillus cereus*, and *Staphylococcus aureus*. Host range testing revealed that vB_EcoS_HSE2 was highly specific. The phage could only infect six of the tested *E*. *coli* strains, but not the other tested *E*. *coli* strains and strains from other species (Table 1). Among the six *E*. *coli* strains sensitive to phage vB_EcoS_HSE2, strain 40199 was isolated from infected wound secretion of the patient, and the other strains were isolated from the urine samples of symptomatic patients. The antibiotic resistance analysis of the phage sensitive strains revealed that all the strains were resistant to more than ten of the tested antibiotics (Table 1).

**Table 1:** Antibiotic resistance and phage sensitivity of strains use in this study.

Strains	Antibiotic Resistance^a^	Phage Sensitivity
*E*. *coli* 40241	AMP^R^, AMC^I^, ATM^R^, PRL^R^, TZP^S^, KZ^R^, CXM^R^, CTX^R^, CRO^R^, CAZ^R^, FEP^R^, FOX^R^, SCF^I^, CIP^R^, LEV^R^, IPM^S^, MEM^S^, SXT^R^, MH^I^, F^S^, AK^S^, CN^S^	S
*E*. *coli* 40199	AMP^R^, AMC^R^, ATM^R^, PRL^R^, TZP^I^, KZ^R^, CXM^R^, CTX^R^, CRO^R^, CAZ^R^, FEP^R^, FOX^I^, SCF^I^, CIP^R^, LEV^R^, IPM^S^, MEM^S^, SXT^S^, MH^I^, F^S^, AK^I^, CN^S^	S
*E*. *coli* 40494	AMP^R^, AMC^R^, ATM^R^, PRL^R^, TZP^I^, KZ^R^, CXM^R^, CTX^R^, CRO^R^, CAZ^R^, FEP^R^, FOX^R^, SCF^R^, CIP^R^, LEV^R^, IPM^R^, MEM^R^, SXT^R^, MH^R^, F^I^, AK^R^, CN^R^	**S**
*E*. *coli* 40498	AMP^R^, AMC^R^, ATM^R^, PRL^R^, TZP^I^, KZ^R^, CXM^R^, CRO^R^, CAZ^R^, FEP^R^, FOX^R^, SCF^R^, CIP^R^, LEV^R^, IPM^R^, MEM^R^, SXT^R^, MH^R^, F^S^, AK^R^, CN^R^	S
*E*. *coli* 40371	AMP^R^, AMC^I^, ATM^R^, PRL^R^, TZP^S^, KZ^R^, CXM^R^, CTX^R^, CRO^R^, CAZ^R^, FEP^R^, FOX^I^, SCF^R^, CIP^R^, LEV^R^, IPM^S^, MEM^S^, SXT^R^, MH^S^, F^S^, AK^S^, CN^R^	S
*E*. *coli* 40372	AMP^R^, AMC^S^, ATM^S^, PRL^R^, TZP^S^, KZ^R^, CXM^R^, CTX^R^, CRO^R^, CAZ^S^, FEP^R^, FOX^S^, SCF^S^, CIP^R^, LEV^R^, IPM^S^, MEM^S^, SXT^S^, MH^S^, F^S^, AK^S^, CN^S^	**S**
*E*. *coli* 40482	AMP^R^, AMC^I^, ATM^R^, PRL^R^, TZP^I^, KZ^R^, CXM^R^, CTX^S^, CRO^R^, CAZ^I^, FEP^R^, FOX^I^, SCF^I^, CIP^R^, LEV^R^, IPM^S^, MEM^S^, SXT^S^, MH^S^, F^S^, AK^S^, CN^S^	R
*E*. *coli* 40311	AMP^R^, AMC^I^, ATM^S^, PRL^S^, TZP^S^, KZ^I^, CXM^I^, CRO^S^, CAZ^S^, FEP^S^, FOX^S^, SCF^S^, CIP^S^, LEV^S^, IPM^S^, MEM^S^, SXT^S^, MH^S^, F^S^, AK^S^, CN^S^	R
*E*. *coli* 40492	AMP^R^, AMC^S^, ATM^S^, PRL^I^, TZP^S^, KZ^I^, CXM^S^, CTX^S^, CRO^S^, CAZ^S^, FEP^S^, FOX^S^, SCF^S^, CIP^S^, LEV^S^, IPM^S^, MEM^S^, SXT^S^, MH^S^, AK^S^, CN^S^	R
*E*. *coli* 40396	AMP^R^, AMC^R^, ATM^R^, PRL^R^, TZP^R^, KZ^R^, CXM^R^, CTX^R^, CRO^R^, CAZ^R^, FEP^R^, FOX^S^, SCF^S^, CIP^R^, LEV^R^, IPM^S^, MEM^S^, SXT^R^, MH^S^, AK^R^, CN^R^	R
*E*. *coli* 40309	AMP^R^, AMC^R^, ATM^R^, PRL^R^, TZP^R^, KZ^R^, CXM^R^, CRO^R^, CAZ^R^, FEP^R^, FOX^R^, SCF^S^, CIP^R^, LEV^R^, IPM^S^, MEM^S^, SXT^S^, MH^I^, F^S^, AK^S^, CN^R^	R
*Klebsiella spp*. 1025	AMP^R^, AMC^R^, ATM^R^, PRL^R^, TZP^R^, KZ^R^, CXM^R^, CTX^R^, CRO^R^, CAZ^R^, FEP^R^, FOX^R^, SCF^R^, CIP^R^, LEV^R^, IPM^R^, MEM^R^, SXT^S^, MH^S^, AK^R^, CN^R^	R
*P*. *aeruginosa* 6312	ATM^S^, FEP^R^, TZP^S^, AK^S^, LEV^R^, IPM^S^, MEM^S^, PB^S^, CN^S^, CAZ^S^, PRL^S^, TOB^S^, SCF^S^, CIP^S^	R
*P*. *aeruginosa* PAO1	NA^b^	R
*S*. *aureus* Sau01	NA	R
*N*. *gonorrhoeae* 6121	NA	R
*S*. *enterica serovar* Typhimurium LT2	NA	R
*B*. *cereus* 6112	NA	R

^a^R, resistant; S, susceptible; I, intermediate. AMP, Ampicillin (10 μg); AMC, Amoxicillin-clavulanate (20/10 μg); ATM, Aztreonam (30 μg); PRL, Piperacillin (100 μg); TZP, Piperacillin-tazobactam (100/10 μg); KZ, Cefazolin (30 μg); CXM, Cefuroxime (30 μg); CTX, Cefotaxime (30 μg); CRO, Ceftriaxone (30 μg); CAZ, Ceftazidime (30 μg); FEP, Cefepime (30 μg); FOX, Cefoxitin (30 μg); SCF, Cefoperazone-sulbactam (75/30 μg); CIP, Ciprofloxacin (5 μg); LEV, Levofloxacin (5 μg); IPM, Imipenem (10 μg); MEM, Meropenem (10 μg); SXT, Trimethoprim (5 μg); MH, Minocycline (30 μg); F, Nitrofurantoin (300 μg); AK, Amikacin (30 μg); CN, Gentamicin (10 μg); TOB, Tobramycin (10 μg); PB, Polymyxin B (300 IU).

^b^The data was Not Available.

### High thermal and pH stability

The thermal and pH tolerance of the phage was analyzed. The phage was highly thermal tolerant, and no significant decrease in phage viability was observed by treating the phage with temperatures below 50 °C for 30 min, while by treating the phage at 60 °C for 30 min, the viability of the phage decreased sharply, and only 26.7% of the phage was alive (Fig. 1D). The phage lost its infective ability after been treated at 70 °C for 30 min. The phage also showed high resistance against pH treatment (Fig. 1E). The phage was stable when treating the phage with pH between 3 and 9, while treating the phage with pH 11, resulting in the phages losing their infective ability.

### Generation of phage resistant mutant strain to phage vB_EcoS_HSE2

**Figure 2:**
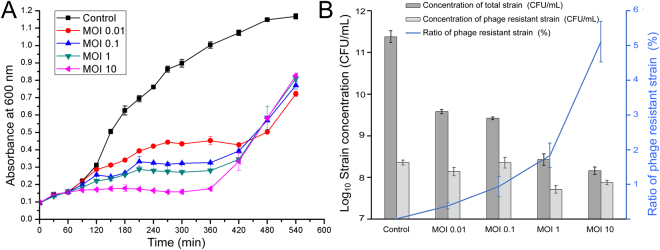
Control effect of phage vB_EcoS_HSE2 to the pathogenic *E*. *coli* strain 40371. (**A**) Growth curve of strain 40371 co-cultivated with phage vB_Ecos_HSE2. The strains were treated with the phage of different MOI and strain without treating by phage was used as control. (**B**) Counts of the total strains and phage resistant strains. The total strain concentrations and concentrations of phage resistant strains were tested after treating the strain with different concentrations of phage for 6 hours. The ratios of phage resistant strain were analyzed by calculating the ratio of phage resistant strains to the total strains.

Due to the co-evolution of phage and host bacterium, phage-resistant bacteria were generated at high frequency, which would influence the efficiency of phage therapy^11^. In this study, the generation of phage resistant bacteria was analyzed. By co-incubating the phage with the strain, the phage significantly inhibited the growth of the bacteria during the first 7 hours (Fig. 2A). However, after that, the turbidity of the cultures increased fast, which might be due to the generation of cell debris or the growth of phage-resistant strains. To figure out the reason for the increased turbidity, the numbers of the viable cells in each culture were determined. The result showed that the final strain concentrations in the cultures treated with phage at an MOI of 0.001, 0.1, 1, and 10, were approximately 1.43%, 0.98%, 0.011%, and 0.0056%, respectively, compared to cultures that have not been treated by phage, suggesting that the phage effectively control the growth of pathogenic *E*. *coli* (Fig. 2B). The micro-examination of the cultures also showed that the phage treated cultures contain fewer bacterial cells, but more cell debris (data not shown). The treatment of the bacterium using phage also reduced the number of phage resistant strains in the culture. For example, the phage-resistant strains in the culture treated by the phage of MOI 1 was 30.9% compared to that in the culture without treatment by phage.

### General features of vB_EcoS_HSE2 genome

**Figure 3:**
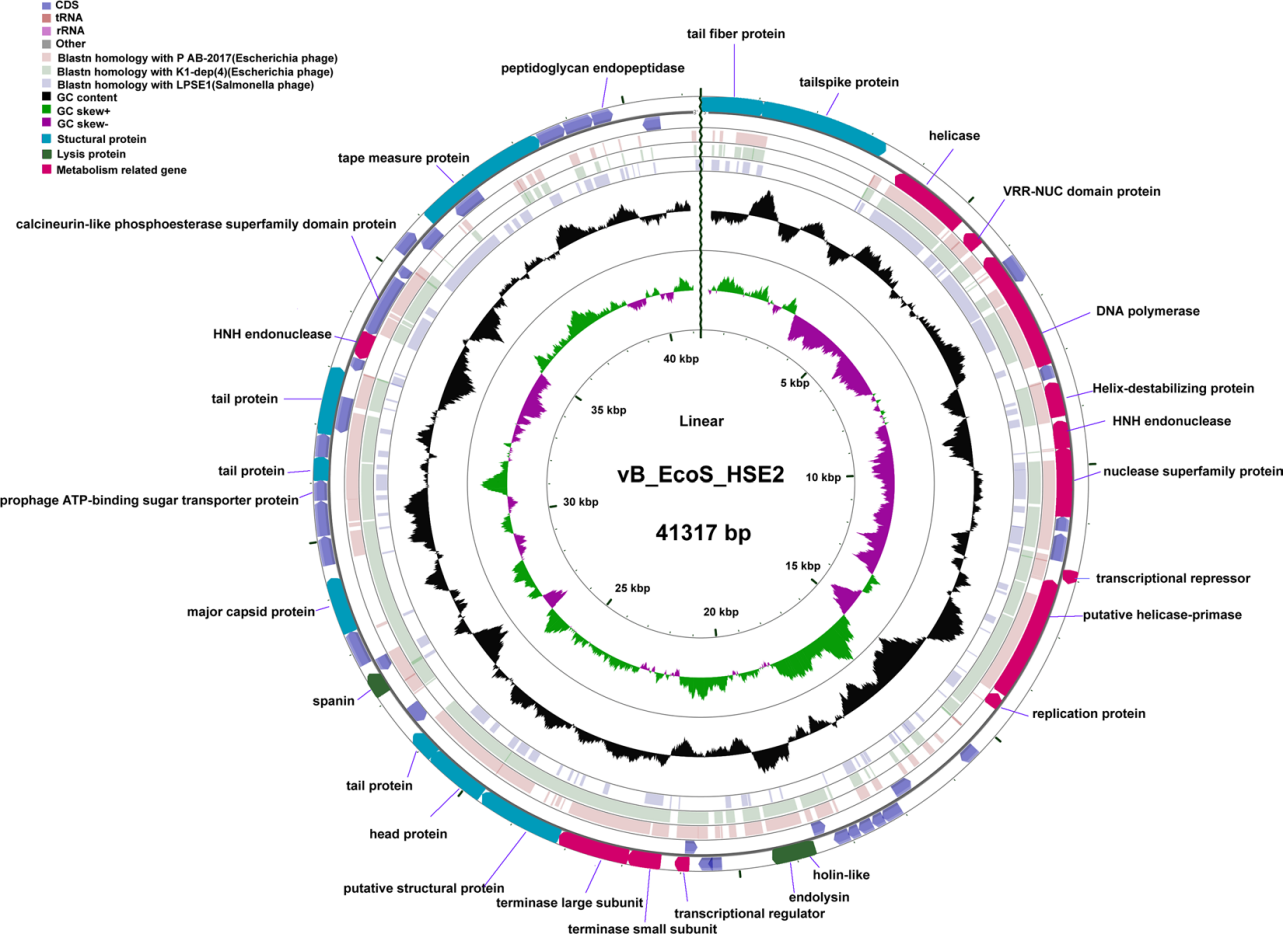
Genome structure of phage vB_EcoS_HSE2 and comparative genomic analysis with phage P AB-2017, K1-dep(4) and LPSE1. The outermost ring presented the CDSs of the linear vB_EcoS_HSE2 genome (blue) and the adjacent three rings showed BLASTN homology between vB_EcoS_HSE2 and P AB-2017 (pale pink), K1-dep(4) (pale green), and LPSE1(pale blue). The middle ring indicated the GC content (black), and the innermost ring represented the GC skew of vB_EcoS_HSE2 genome. The predicted functions of the CDSs were indicated.

The genome of phage vB_EcoS_HSE2 was a linear genome with a genome size of 41,371 bp. The G + C content of the genome was 50.87%, which was almost the same as the genome of the *E*. *coli* strains (GenBank accession number NC_002695.1). Fifty-eight open reading frames (ORFs), including 24 functionally annotated ORFs, were predicted in the phage genome. No tRNAs and repeat sequences were found in the genome. The ORFs were mainly annotated as DNA metabolism associated proteins, cell lysis related proteins, and structural proteins (Fig. 3). Based on the locations of the functional genes, the genome of phage vB_EcoS_HSE2 showed modular genome structure and genes with associated functions were mainly located in the same gene cluster.

### Numerous nucleotide metabolism associated genes

At least eleven genes encoded by phage vB_EcoS_HSE2 were predicted to play roles in the process of phage nucleotide metabolism, including a helicase, a helicase-primase, a DNA polymerase, a nuclease superfamily protein, a transcriptional repressor, a transcriptional regulator, a replication protein, a VRR-NUC domain protein, a helix-destabilizing protein, and two alleles of HNH endonuclease (Fig. 3). The highly present of the DNA metabolism associated genes in vB_EcoS_HSE2 genome might reduce the dependence of the phage nucleotide metabolism on the host bacterium.

### Three component host lysis system

Three genes that might be involved in the lysis of the bacterial cell were found in phage vB_EcoS_HSE2 genome. Gene *gp*24 and *gp*25, encoding holin-like protein and endolysin, respectively, might form a holin-dependent phage host lysis system to lyse the host cell at the end of the phage life cycle to release progeny phage virions^12^. According to previous reports, the spanin complex conducts the final step in host lysis by disrupting the outer membrane after holin and endolysin have permeabilized the inner membrane and degraded the host peptidoglycans, respectively^13^. One spanin protein encoding gene (*gp*36) was found located downstream of the phage host lysis system and might facilitate the release of the phage virion from the out membrane.

### Mosaic structure of phage vB_EcoS_HSE2 structural proteins

Totally, twelve ORFs in phage vB_EcoS_HSE2 genome were found to encode phage structural proteins and these genes were scattered in the genome (Fig. 3). These eleven ORFs mainly encode proteins that function as terminases (Gp30 and Gp31), tail proteins (Gp01, Gp02, Gp34, Gp43, Gp45, Gp53), head proteins (Gp33 and Gp39), and two putative structural proteins (Gp32 and Gp35). In additional to the protein Gp01, Gp02, and Gp53, the other annotated structural proteins showed similarities that were higher than 95% with structural proteins of the other *E*. *coli* phages.

**Figure 4:**
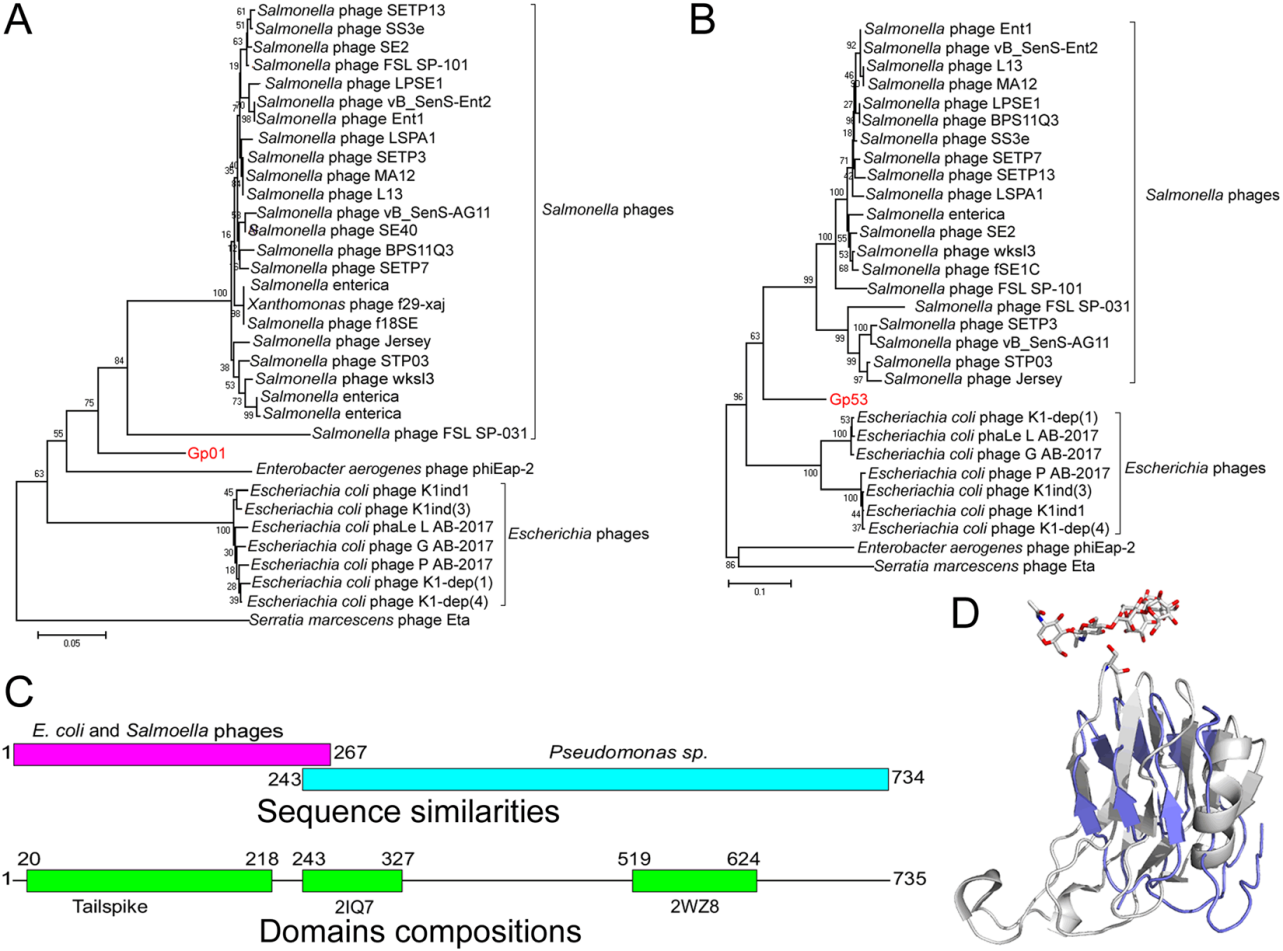
Phylogenetic and functional analysis of the phage structural proteins. (**A**) and (**B**) Phylogenetic analysis of protein Gp01 and Gp53. The proteins that showed similarity with protein Gp01 and Gp53 were collected from GenBank and used for phylogenetic tree construction using Mega6.0 with neighbor-joining method with the bootstrap replicate of 1000. (**C**) Schematic diagram of the similarity and domain composition of Gp02. The N-terminus and C-terminus were similar with *E*. *coli* phages and *Pseudomonas* sp. proteins, respectively. The protein Gp02 contained three functional predicted domains, including the domain similar to the tailspike protein, endopolygalacturonase (indicated as PDB entry 2IQ7), and cellulosome protein dockerin carbohydrate binding module (indicated as PDB entry 2WZ8). (**D**) Protein structure modeling of residues 243–327 from protein Gp02. The structure of the protein endopolygalacturonase (PDB entry 2IQ7) was used as template for structure modeling and the polysaccharide was indicated.

BLASTP analysis of Gp01 reveals that the protein showed highest similarity with the tail fiber proteins of *Salmonella* phages (Fig. 4A), including the *Salmonella* phage SS3e, which shows extremely broad host spectrum against strains of various *Salmonella* serovars, *E*. *coli*, *Shigella sonnei*, *Enterobacter cloacae*, and *Serratia marcescens*^14^ and the gene might be obtained from the *Salmonella* phage genome by horizontal gene transfer during their co-infection of the *Salmonella* strain or *E*. *coli* strain. BLASTP analysis of GP02 showed that the protein had a mosaic structure. The N-terminal 267 amino acids (from residues 1–267) of the protein showed high similarities with the tail proteins of *Escherichia* phage K1ind3 (42% similarity) and *Escherichia* phage G AB2017 (68% similarity), while the 492 amino acids at the C-terminus (from residues 243–734) only showed similarities with proteins of *Pseudomonas sp*. strain (41% similarity) and *Neisseria* sp. strain (37% similarity), but not the protein from the *E*. *coli* phage (Fig. 4C). Functional prediction of the C-terminus of protein Gp02 revealed that the residues between 243–327 were similar to the endopolygalacturonase (PDB entry 2iq7) in structure^15^ and contained the polysaccharide ligand binding residues (Fig. 4D). The residues between 519–624 were similar with the cellulosome protein dockerin carbohydrate binding module (PDB entry 2wz8) in structure^16^. Several phages infect the host bacteria by binding to the polysaccharides on the bacterial cell surface and some phages also contain virion-associated enzymes to overcome the carbohydrate barriers during infection^17,18^. The containment of polysaccharide binding domain in the phage structural might facilitate the infection of the phage.

Except for *gp*02, the gene *gp*53, which encodes the tape measure protein, was the longest gene in vB_EcoS_HSE2 genome and also had a mosaic nature. The tape measure protein dictates the tail length and facilitates DNA transit to the bacterial cell cytoplasm during infection^19–21^. BLASTN analysis of *gp*53 reveals that the 3′ termini 610-bp of the gene showed the highest similarity (83%) with the tape measure protein gene of *Escherichia* phages, while the 5′ terminal 1577-bp terminus of the gene showed the highest similarity (78%) with the tape measure protein gene of *Salmonella* phages. BLASTP analysis of Gp53 revealed that the protein showed the highest similarity (82% similarity) with the *Salmonella* phage SETP7, while the highest similarity of thisprotein to protein encoded by the *E*. *coli* phage is 55% (*E*. *coli* phage P AB-2017). Phylogenetic analysis of Gp53 showed that the protein was close in evolution with the tape measure proteins from the *Salmonella* phages. The results indicated that the gene *gp*53 might also have encountered horizontal gene transfer and gene recombination during the evolutionary process of the phage.

### Phylogenetic and comparative genomic analysis of phage vB_EcoS_HSE2

**Figure 5:**
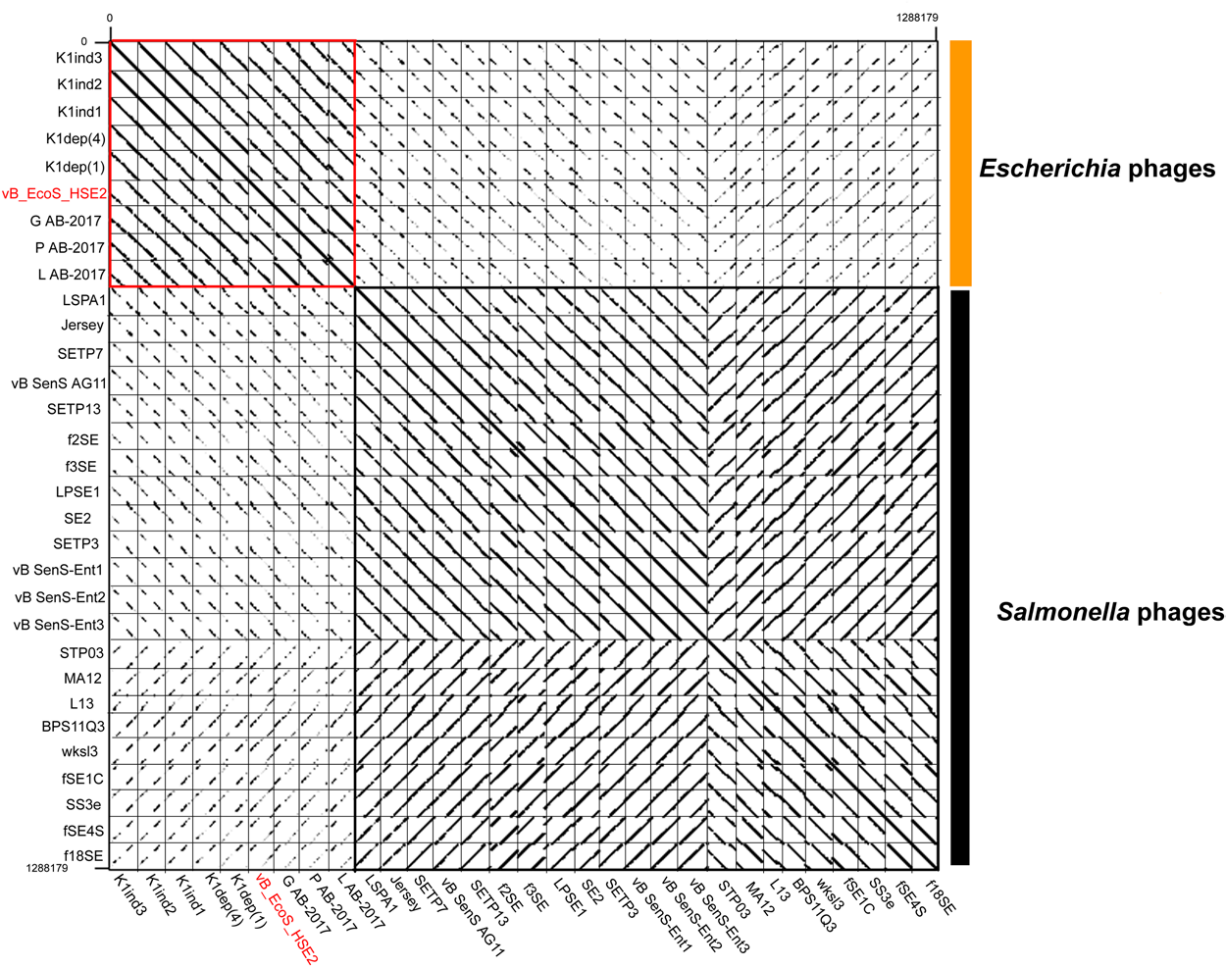
Comparative genomic analysis of phages that were similar with phage vB_EcoS_HSE2. The phages with genome similarity higher than 20% were collected from GenBank and used for dot plot analysis. The dot plot analysis was performed by using Gepard.

**Figure 6:**
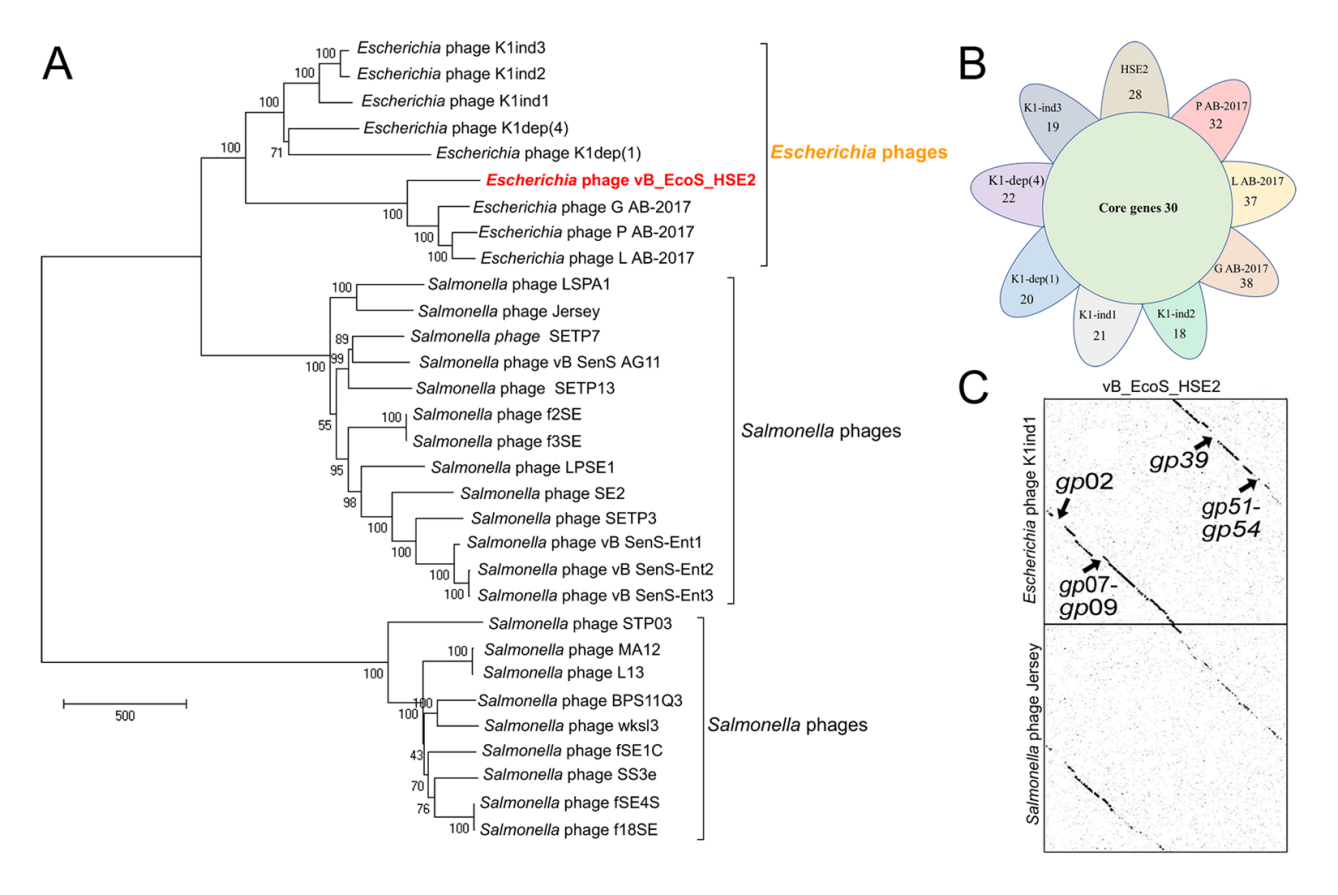
Phylogenetic and comparative genomic analysis of the phage vB_EcoS_HSE2. (**A**) Phylogenetic analysis of phages that showed similarity with the phage vB_EcoS_HSE2. The phage genomes that showed similarity higher than 20% with the genome of phage vB_EcoS_HSE2 were collected and used for phylogenetic tree construction. (**B**) Core-genome analysis of phages from the same evolutionary branch with phage vB_EcoS_HSE2. The numbers of core genes for the 9 phages were indicated in the central circle, and the number of specific genes were also shown. (**C**) Dot plot analysis of phage vB_EcoS_HSE2 with phage K1ind1 and Jersery. The genes that were absent in phage K1ind1 genome are shown.

BLASTN analysis of the phage vB_EcoS_HSE2 genome showed that the genome of phage vB_EcoS_HSE2 was highly similar (similarity approximately 50%) with the genomes of the *E*. *coli* phage K1H, K1ind2, K1G, K1ind3, L AB-2017, P AB-2017, and G AB-2017. The phage also showed similarity with *Salmonella* phages, such as phage BPS11Q3, Jersey, and SS3e (Fig. 5). Phylogenetic analysis of the phage vB_EcoS_HSE2 revealed that though the phage was close in relationship with the *E*. *coli* phages, it was clustered into the single branch (Fig. 6A). The core genes of the nine phages closely in evolution were analyzed and the result showed that 30 proteins were found to be conserved in all the six phages (Fig. 6B). The functions of these proteins were mainly related to the phage structure proteins and DNA replication, as well as host lysis, which were essential for the phage life cycle (Table S2).

Based on the phylogenetic analysis of phage vB_EcoS-HSE2, the genomes of *E*. *coli* phage K1ind1 and *Salmonella* phage Jersey, accompany the genome of phage vB_EcoS_HSE2, were used for comparative genomic analysis (Fig. 5D). The result of the comparative genomic analysis showed that the genome of phage vB_EcoS_HSE2 showed a highly co-linear relationship with the genomes of phage K1ind1 and Jersey, except for the deletion of gene *gp*02, *gp*07-gp09, *gp*39, and *gp*51-*gp*53. Meanwhile, the genes *gp*07-gp09 and genes *gp*51-*gp*53, which were absent in phage K1ind1 genome, show homologues with the genes of the *Salmonella* phage Jersey.

## Discussion

The outbreak of multidrug resistance *E*. *coli* has greatly limited antibiotic therapy and made it urgent to develop novel control strategies. The phage regained attention for its’ specific lytic activity and special control mechanisms against pathogenic bacterium. Numerous phages infecting *E*. *coli* have been isolated and their potential in controlling pathogenic multidrug resistant *E*. *coli* strains have been evaluated^22–24^. However, the generation of phage resistance was even faster than the generation of antibiotic resistance and, as described in this study, the phage-resistant bacteria even existed before the use of phage preparation. Construction of phage cocktail containing phages of different genetic backgrounds could efficiently reduce the generation of phage resistance and increase the efficiency of phage therapy. Thus, it is valuable to isolate phages with novel genetic backgrounds. In this study, an *E*. *coli* phage vB_EcoS_HSE2 that could efficiently control pathogenic *E*. *coli* by reducing both the cell concentrations of phage sensitive bacterium and the phage resistant bacterium was isolated and characterized. The phage exhibited highly specific host range and could only infect partial of the tested *E*. *coli* strains, but not the other *E*. *coli* strains or strains from the other species. Besides, no toxin gene, antibiotic resistance gene, phage lysogeny factor and other pathogen-related genes were found in the phage genome, suggesting that the phage was biosafe for being used in control pathogenic *E*. *coli*.

Comparative genomic analysis revealed that, though the phage showed the highest similarity with *E*. *coli* phage, several of its structural proteins, especially the tail proteins (Gp01 and Gp53), were similar with that of *Salmonella* phages. According to previous reports, the tail fiber protein and tail spike protein of *Salmonella* phage shows high diversity due to the gene recombination at the C-termini^25–27^. The tail fiber and tail spike are appendages in the phage tail that facilitates the initial binding of the phage to the bacterial host and have roles in host specificity determination^28^. As shown in our study, highly variable tail fiber protein typically presented a fairly conserved N-terminus, with low identities in the C terminus. The conservations of the N-terminal residues were consistent with the fact that this region attaches to the baseplate, while the remainder of the protein projects from the surface of the phage tail distal and appears to be involved in the initial binding to the host. The gene *gp*02, which was located immediately after gp01, was found to be highly similar to the genes from strains of *Pseudomonas sp*. and *Neisseria* sp., which are both human pathogens^29,30^. Gp02 was the phage tail spike protein and functional analysis of Gp02 revealed that the C-termini of the protein contained a polysaccharide binding domain that might work as a phage receptor binding protein. Further functional analysis of these two proteins and the interaction between the phage vB_EcoS_HSE2 and the host bacterium might figure out the roles of these two proteins in phage host range determination.

The genes *gp*01 and *gp*02 were located at the very beginning of the 5′-termini of the phage genome, and the genes downstream of these two genes were transcribed in different directions as these two genes, suggesting that these two genes might be obtained by genome recombination. The horizontal gene transfer is one of the most frequently approach for microbial genome mutation and the phage was thought to be the most important vector to delivery horizontal gene transfer^31,32^. In consideration of the high similarity of *gp*01 with genes of *Salmonella* phages and the high similarity of *gp*02 with genes *Pseudomonas sp*. and *Neisseria* sp., it is rational to speculate that the phage had undergone at least two genome recombination events during its evolutionary process. The 3′-termini of gene *gp*02 might be obtained from *Pseudomonas sp*. or *Neisseria* sp. during infection of the ancestral phage to one of these two strains. Subsequently, or antecedently, the recombinant phage co-infected with the other *Salmonella* phages obtained the gene *gp*01. Except for the gene *gp*01 and *gp*02, the gene *gp*53, which was located near the 3′-termini of the phage genome, also showed low similarity with the *E*. *coli* phage, but highly similar 3′-termini with the genes of *Salmonella* phages. The results indicating that the 3′-termini of the phage gene *gp*53 might also be acquired from *Salmonella* phages by genome recombination during co-infection with *Salmonella* phages. In summary, during the evolutionary process, the phage vB_EcoS_HSE2 underwent at least three genome recombination events by at least two infection processes to the non-*E*. *coli* strains. The phage was isolated from hospital sewage, which contained several pathogenic bacteria, including strains of *E*. *coli*, *Pseudomonas sp*., *Neisseria* sp., and *Salmonella* sp., which provided benefits for the recombination of the phage.

In conclusion, this study isolated a phage that might be used for pathogenic *E*. *coli* control. Genomic analysis of the phage revealed that the phage had undergone at least three rounds of genome recombination events during the evolutionary process. The finding of this study not only provides resources for developing phage therapy against *E*. *coli*, but also showed the highly variable genome structure of the phage.

## Materials and Methods

### Strain and antimicrobial susceptibility test

The *E*. *coli* strains used in this study were isolated from urine or wound secretion of symptomatic patients in Liyuan Hospital (Wuhan, China) and stored by our laboratory. Antimicrobial susceptibility tests of these strains were conducted using the Kirby-Bauer disk diffusion method according the Clinical and Laboratory Standards Institute (CLSI) guidelines 2017^33^. *E*. *coli* strains were cultured in Luria-Bertani (LB) broth medium at 37 °C. *E*. *coli* strain 40371 was used as an indicator strain for phage isolation.

### Phage isolation and propagation

Hospital sewage samples were collected from Liyuan hospital and were centrifuged at 12,000 × g for 10 min to remove the solid impurities. The supernatants were filtered through a 0.22-μm pore-size membrane filter to remove bacterial debris. For each sample, 200 μL of supernatant was added into 4 ml exponential growth strain 40371 and incubated for additional 8 h. After incubation, the supernatant of the culture was collected by centrifugation at 8,000 × g at 4 °C for 30 min and filtered through a 0.22-μm pore-size membrane filter. Then, 100 μl of the filtered supernatant was mixed with 200 μl of exponential growth 40371. After binding for 10 min, the mixture was added into 4 ml of melt semisolid LB medium and then overlaid onto an LB agar plate. Phage propagation, purification, and the efficiency-of-plating test were carried out by double-agar overlay assay as previously described^34^.

### Electron microscopy observation of phage virions

To prepare phage for transmission electron microscopy (TEM) observation, the purified phage suspension was deposited on the cuprum grid with carbon-coated Formvar film and stained with 2% potassium phosphotungstate (pH 7.2). After air drying, the sample was observed with a TEM (H-7000FA; Hitachi, Tokyo, Japan) at an acceleration voltage of 100 kV.

### Host range determination

The lytic activity of vB_EcoS_HSE2 was tested against 13 species as determined by standard spot tests^35^. The test strains were grown overnight in LB broth and 200 μL of the strain was mixed with 4 ml melt semisolid medium to pour the double-layer medium. Then, 1 μL purified phage suspensions (approximately 10^8^ Pfu/ml) were spotted onto the upper layer medium and left to incubate overnight. Bacterial sensitivity to phage coincided with the formation of the spot where the phage suspension was deposited. Each strain was tested three times at 37 °C.

### Phage stability assay

The purified phage suspensions were stored at −80, 4, and 30 °C, respectively, and the phage titer was determined by double-agar overlay assay immediately and every week after storage^36^. For the thermal stability assay, equal volumes of phage (approximately 10^8^ Pfu/ml) were added into the 1.5 ml tube and incubated at 28 °C, 37 °C, 50 °C, 60 °C, and 70 °C for 30 min. After treatment, the tube was cooled slowly and placed in an ice water bath, and then these samples were assayed to determine the surviving phages. For the pH stability assay, after treating the phage at a pH of 3.0, 5.0, 7.0, 9.0, and 11.0 for 30 min, the phage suspensions were used for phage titer determination immediately after the finish of the treatment. The results were expressed as a percentage of the initial viral counts. Each assay was performed as three repetitions and the values represented are the means.

### One-step growth curve analysis

To analyze the one step growth curve of the phage, phage was added into the exponential growth strain at an MOI of 1.0 at 37 °C for 5 min for phage adsorption. The non-adsorbed phage was removed by centrifugation at 10,000 × g for 1 min, and the pellet was resuspended in 50 ml of LB broth. The phage titer in the culture was determined every 10 min. The burst size of the phage was determined as previously described^37^.

### Assay of the appearance ratio of phage resistant bacteria

To determine the phage-resistant ratio of the strain, the exponential growth strains were cultivated with the phage with a multiplicity of infection (MOI) of 0.01, 0.1, 1, and 10, and added into LB broth at a ratio of 1%. Cultures that had not been added phage were used as control. The strains were incubated at 37 °C with moderate shaking for 6 hours and used for phage-resistant strain counting. To count the number of phage resistant bacterial cells, the phage was mixed with the upper semisolid medium with a final concentration of 10^8^ PFU/ml, which could inhibit the growth of the sensitive strain, and poured onto the plate. The strain cultures were gradient diluted and spread onto the upper medium. After overnight cultivation at 37 °C, the concentration of the phage-resistant strain were recorded and calculated. The gradient diluted cultures were also spread onto an LB agar plate to determine the total bacteria concentrations.

### Phage genome extraction and sequencing

The purified phage suspension was used for phage genome purification. Phage genome extraction was performed as described before by phenol-chloroform extraction with protease K-sodium dodecyl sulfate (SDS) treatment^38^. The phage genome was sequenced using an Illumina HiSeq. 2500 sequencer. The reads obtained by sequencing were assembled into contigs using SPAdes-3.5.0 software^39^. The coding sequences (CDSs) of the phage genomes were predicted using the FGENE SV0 software (Softberry, http://www.softberry.com/berry.phtml?topic=virus0&group=programs&subgroup=gfindv) and by visual inspection. The putative function of each gene was predicted by performing a search in the National Center for Biotechnology Information nonredundant (NR) database and the CDD databases using the basic local alignment search tool (BLAST)^40^. The motif and functional domain composition of the predicted protein were analyzed by searching the Pfam database and by using HHpred^41,42^. Phage genome annotation was visualized by using CGview^43^. The genes encoding the putative tRNAs were analyzed using tRNAScan^44^. Tandem repeat and insert sequences in the phage genome were analyzed by using Tandem Repeat Finder and Repeat Masker^45,46^. Comparative genome analysis of the phages was carried out using Gepard 1.3^47,48^. Core gene analysis of phages was performed by CoreGenes 3.0 and genes with scores above 75 were regarded as the core genes^49^. The phylogenetic trees in the present study were constructed using Mega 6.0^50^.

### Nucleotide sequence accession number

The nucleotide sequence of the phage vB_EcoS_HSE2 genome was deposited in GenBank under the accession number MG252615.

## Electronic supplementary material

Supplementary information
