# Supplementary material for: Characterization of a newly isolated phage infecting pathogenic Escherichia coli and analysis of its mosaic structural genes
Source: Sci Rep. 2018 May 24;8:8086. doi: 10.1038/s41598-018-26004-4 (PMC5967307; doi:10.1038/s41598-018-26004-4)
Supplement: Supplementary file 1 — Supplementary information [file 41598_2018_26004_MOESM1_ESM.pdf]

Characterization of a newly isolated phage infecting pathogenic *Escherichia coli* and analysis of its mosaic structural genes

Qin Peng[1], Yihui Yuan[2,*]

[1] Ministry of Education Key Laboratory for Ecology of Tropical Islands, College of Life Sciences, Hainan Normal University, Haikou 570100, P. R. China

[2] State Key Laboratory of Marine Resource Utilization in South China Sea, Hainan University , Haikou 570228, China.

*Correspondence and requests for materials should be addressed to Y. Y. (yyhheda@126.com).

Table S1. Genome annotations of phage vB_EcoS_HSE2.

Gene accession	Direction	Nucleotide position		Length (amino acid)	Function	Best match	Similarity	E-value
		start	end					
gp01	+	11	1093	360	tail fiber protein	*Salmonella* phage STP03 orf2	83%	0
gp02	+	1106	3313	735	tailspike protein	*Salmonella* phage SE40 protein ARK07384.1	11.80%	2.00E-42
gp03	-	5139	3715	474	helicase	*Escherichia* phage L AB-2017 orf64	99%	0
gp04	-	5651	5367	94	VRR-NUC domain protein	*Escherichia* phage L AB-2017 orf62	97%	4.00E-61
gp05	-	8136	5932	734	DNA polymerase	*Salmonella* phage ST4 proteinAFO70776.1	89.10%	0
gp06	+	6158	6634	158	hypothetical protein	No hits		
gp07	-	8411	8133	92	hypothetical protein	*Escherichia* phage vB_EcoP_SU10 orf55	57%	7.00E-26
gp08	-	9097	8471	208	Helix-destabilizing protein	*Escherichia* phage K1-dep(4) orf39	99%	2.00E-151
gp09	-	9675	9184	163	HNH endonuclease	*Enterobacter* phage phiEap-2 orf60	49.50%	4.00E-48
gp10	-	10913	9672	413	nuclease superfamily protein	*Escherichia* phage G AB-2017 orf58	89%	0.00E+00
gp11	-	11182	10910	90	hypothetical protein	*Salmonella* phage Jersey orf47	97%	1.00E-60
gp12	-	11726	11226	166	hypothetical protein	*Salmonella* phage f18SE orf3	74%	2.00E-71
gp13	+	11851	12066	71	transcriptional repressor	*Escherichia* phage L AB-2017 orf53	99%	3.00E-41
gp14	-	14322	12085	745	putative helicase-primase	*Escherichia* phage K1ind1 orf35	94%	0
gp15	-	14614	14381	77	replication protein	*Escherichia* phage K1ind1 orf36	95%	3.00E-44
gp16	+	15371	15688	105	hypothetical protein	No hits		
gp17	-	16976	16617	119	hypothetical protein	No hits		

gp18	+	17028	17384	118	hypothetical protein	*Escherichia* phage K1-dep(1) orf40	49%	2.00E-45
gp19	+	17366	17584	72	hypothetical protein	*Salmonella* phage BPS11Q3 orf25	90%	2.00E-40
gp20	+	17587	17841	84	hypothetical protein	*Escherichia* phage G AB-2017 orf39	52%	6.00E-14
gp21	+	17841	18044	67	hypothetical protein	*Escherichia* phage phAPEC8 orf36	48.90%	6.00E-12
gp22	+	18028	18309	93	hypothetical protein	*Escherichia* phage K1ind1 orf39	87%	1.00E-53
gp23	-	18605	18360	81	hypothetical protein	No hits		
gp24	+	18686	18958	90	holin-like	*Escherichia* phage G AB-2017 orf36	96%	4.00E-57
gp25	+	18921	19421	166	endolysin	*Escherichia* phage K1-ind(3) orf41	94%	5.00E-111
gp26	+	20295	20534	79	hypothetical protein	*Escherichia* phage K1ind1 orf44	72%	1.00E-33
gp27	+	20455	20703	82	hypothetical protein	*Escherichia* phage L AB-2017 orf30	70.81%	2.00E-35
gp28	-	20943	20719	74	hypothetical protein	No hits		
gp29	+	20873	21109	78	transcriptional regulator	*Escherichia* phage K1-dep(4) orf48	100%	5.00E-51
gp30	+	21368	21928	186	terminase small subunit	*Escherichia* phage K1-ind(3) orf46	98%	1.00E-130
gp31	+	21925	23175	416	terminase	*Escherichia* phage K1-dep(1) orf1	99%	0.00E+00
gp32	+	23187	24680	497	putative structural protein	*Escherichia* phage L AB-2017 orf24	97%	0.00E+00
gp33	+	24717	25793	358	head protein	*Escherichia* phage K1-ind(2) orf3	96%	0.00E+00
gp34	+	25793	26254	153	tail protein	*Escherichia* phage K1-ind(3) orf5	94%	6.00E-98
gp35	-	26695	26351	114	hypothetical protein	No hits		3.00E-19
gp36	+	27168	27554	128	spanin	*Escherichia* phage K1-ind(3) orf6	95%	2.00E-82
gp37	-	27738	27469	89	hypothetical protein	No hits		
gp38	+	27743	28369	208	hypothetical protein	*Escherichia* phage G AB-2017 orf20	100%	2.00E-145
gp39	+	28376	29344	322	major capsid	*Escherichia* phage	97%	0

					protein	K1-dep(1) orf8		
gp40	+	29573	30085	170	hypothetical protein	*Escherichia* phage K1-ind(3) orf13	95%	3.00E-112
gp41	+	30088	30702	204	hypothetical protein	*Escherichia* phage L AB-2017 orf15	91%	7.00E-129
gp42	+	30702	31061	119	prophage ATP-binding sugar transporter protein	*Escherichia* phage G AB-2017 orf16	96%	7.00E-78
gp43	+	31058	31453	131	tail protein	*Escherichia* phage K1-ind(3) ORF15B	98%	2.00E-88
gp44	+	31450	31866	138	hypothetical protein	*Escherichia* phage K1-dep(4) orf16	97%	4.00E-94
gp45	+	31869	33035	388	tail protein	*Escherichia* phage K1-dep(1) orf14B	97%	0
gp46	-	32560	31910	216	hypothetical protein	No hits		
gp47	-	33293	33063	76	hypothetical protein	*Salmonella* phage FSL SP-101 protein SP101_00095	92%	7.00E-46
gp48	-	33777	33307	156	HNH endonuclease	*Salmonella* phage L13 orf2	45.08%	2.00E-33
gp49	-	34904	33774	376	calcineurin-like phosphoesterase superfamily domain protein	*Escherichia* phage P AB-2017 orf12	95.04%	0
gp50	-	35149	34943	68	hypothetical protein	*Escherichia* phage K1-dep(4) orf21	92%	6.00E-36
gp51	+	35302	35718	138	hypothetical protein	*Salmonella* phage L13 orf11	92%	2.00E-89
gp52	-	36043	35615	142	hypothetical protein	No hits		
gp53	+	36076	38412	778	tape measure protein	*Salmonella* phage SETP7 orf26	82%	0
gp54	-	37049	36477	190	hypothetical protein	No hits		
gp55	+	38416	38910	164	hypothetical protein	*Serratia* phage Eta orf64	82%	5.00E-97
gp56	+	38907	39422	171	hypothetical protein	*Salmonella* phage wksl3 protein AFO12377.1	92%	2.00E-114
gp57	+	39419	39784	121	peptidoglycan endopeptidase	*Salmonella* phage SETP3 orf29	79%	2.00E-67
gp58	-	40583	40257	108	hypothetical	No hits		

protein

Table S2. Core genes among nine *Escherichia* phages.

Protein function	HSE2 accession	L AB-2017 accession	G AB-2017 accession	P AB-2017 accession	K1-dep(1) accession	K1-dep(4) accession	K1ind1 accession	K1ind2 accession	K1ind3 accession
tail fiber protein	gp1	AQN31892.1	AQN31759.1	AQN31943.1	ADA82321.1	ADA82272.1	ADA82373.1	ADA82424.1	ADA82473.1
tailspike protein	gp2	AQN31891.1	AQN31758.1	AQN31942.1	ADA82322.1	ADA82273.1	ADA82374.1	ADA82425.1	ADA82474.1
helicase	gp3	AQN31889.1	AQN31823.1	AQN31940.1	ADA82325.1	ADA82275.1	ADA82375.1	ADA82426.1	ADA82475.1
DNA polymerase	gp5	AQN31885.1	AQN31818.1	AQN31996.	ADA82330.1	ADA82280.1	ADA82378.1	ADA82429.1	ADA82478.1
nuclease superfamily protein	gp10	AQN31882.1	AQN31815.1	AQN31993.1	ADA82332.1	ADA82282.1	ADA82379.1	ADA82431.1	ADA82480.1
hypothetical protein	gp12	AQN31879.1	AQN31811.1	AQN31991.1	ADA82334.1	ADA82284.1	ADA82382.1	ADA82432.1	ADA82481.1
putative helicase-primase	gp14	AQN31877.1	AQN31809.1	AQN31989.1	ADA82336.1	ADA82286.	ADA82383.1	ADA82433.1	ADA82482.1
hypothetical protein	gp18	AQN31865.1	AQN31797.1	AQN31982.1	ADA82338.1	ADA82288.1	ADA82386.1	ADA82435.1	ADA82484.1
holin-like	gp24	AQN31861.1	AQN31793.1	AQN31978.1	ADA82341.1	ADA82291.1	ADA82389.1	ADA82438.1	ADA82487.1
endolysin	gp25	AQN31860.1	AQN31792.1	AQN31977.1	ADA82342.1	ADA82292.1	ADA82390.1	ADA82439.1	ADA82488.1
transcriptional regulator	gp29	AQN31853.1	AQN31786.1	AQN31971.1	ADA82344.1	ADA82294.1	ADA82395.1	ADA82443.1	ADA82492.1
DNA-packaging protein	gp30	AQN31851.1	AQN31784.1	AQN31968.1	ADA82345.1	ADA82295.1	ADA82396.1	ADA82444.1	ADA82493.1
terminase	gp31	AQN31850.1	AQN31783.1	AQN31967.1	ADA82296.1	ADA82244.1	ADA82346.1	ADA82397.1	ADA82445.1
putative structural protein	gp32	AQN31849.1	AQN31782.1	AQN31966.1	ADA82297.1	ADA82245.1	ADA82347.1	ADA82398.1	ADA82446.1
head protein	gp33	AQN31848.1	AQN31781.1	AQN31965.1	ADA82298.1	ADA82246.1	ADA82348.1	ADA82399.1	ADA82447.1
tail protein	gp34	AQN31847.1	AQN31780.1	AQN31964.1	ADA82299.1	ADA82247.1	ADA82349.1	ADA82400.1	ADA82449.1
spanin	gp36	AQN31846.1	AQN31779.1	AQN31963.1	ADA82300.1	ADA82248.1	ADA82350.1	ADA82401.1	ADA82450.1

hypothetical protein	gp38	AQN31844.1	AQN31777.1	AQN31961.1	ADA82302.1	ADA82250.1	ADA82352.1	ADA82403.1	ADA82452.1
hypothetical protein	gp40	AQN31841.1	AQN31774.1	AQN31958.1	ADA82305.1	ADA82256.1	ADA82357.1	ADA82408.1	ADA82457.1
hypothetical protein	gp41	AQN31840.1	AQN31773.1	AQN31957.1	ADA82306.1	ADA82259.1	ADA82358.1	ADA82409.1	ADA82458.1
prophage ATP-binding sugar transporter protein	gp42	AQN31839.1	AQN31773.1	AQN31956.1	ADA82307.1	ADA82257.1	ADA82359.1	ADA82410.1	ADA82459.1
tail protein	gp43	AQN31838.1	AQN31772.1	AQN31955.1	ADA82308.1	ADA82258.1	ADA82360.1	ADA82411.1	ADA82460.1
hypothetical protein	gp44	AQN31837.1	AQN31771.1	AQN31954.1	ADA82309.1	ADA82260.1	ADA82361.1	ADA82412.1	ADA82461.1
tail protein	gp45	AQN31836.1	AQN31770.1	AQN31953.1	ADA82310.1	ADA82261.1	ADA82362.1	ADA82413.1	ADA82462.1
calcineurin-like phosphoesterase superfamily domain protein	gp49	AQN31833.1	AQN31767.1	AQN31950.1	ADA82313.1	ADA82264.1	ADA82365.1	ADA82416.1	ADA82465.1
hypothetical protein	gp51	AQN31831.1	AQN31765.1	AQN31948.1	ADA82315.1	ADA82266.1	ADA82367.1	ADA82418.1	ADA82467.1
tape measure protein	gp53	AQN31829.1	AQN31763.1	AQN31947.1	ADA82317.1	ADA82268.1	ADA82369.1	ADA82420.1	ADA82469.1
hypothetical protein	gp55	AQN31828.1	AQN31762.1	AQN31946.1	ADA82318.1	ADA82269.1	ADA82370.1	ADA82421.1	ADA82470.1
hypothetical protein	gp56	AQN31827.1	AQN31761.1	AQN31945.1	ADA82319.1	ADA82270.1	ADA82371.1	ADA82422.1	ADA82471.1
peptidoglycan endopeptidase	gp57	AQN31826.1	AQN31760.1	AQN31944.1	ADA82320.1	ADA82271.1	ADA82372.1	ADA82423.1	ADA82472.1
